# Establishment and evaluation of hematological and serum biochemical reference intervals in Chinese local donkey populations

**DOI:** 10.1186/s12917-026-05556-y

**Published:** 2026-05-18

**Authors:** Qifei Zhu, Muhammad Zahoor Khan, Yadi Jing, Xiaotong Liu, Wenting Chen, Mingyang Geng, Bing Liu, Yunliang Zhang, Shishuai Xing, Yongdong Peng, Changfa wang

**Affiliations:** 1 Liaocheng Research Institute of Donkey High-Efficiency Breeding and Ecological Feeding, College of Agriculture and Biology, Liaocheng University, Liaocheng, 252000 China; 2 Ili Kazak Autonomous Prefecture Livestock General Station, Ili, 835000 Xinjiang China; 3 National Engineering Research Center for Gelatin-based TCM, Dong-E E-Jiao Co., Ltd, 78 E-Jiao Street Donge County, Donge, 252200 Liaocheng, Shandong China; 4 Xinjiang Shanhai Animal Husbandry Co., Ltd, Yili, 835000 Xinjiang China; 5 Yucheng Huimin Agricultural Science and Technology Co., Ltd, Yucheng, 251200 Shandong China

**Keywords:** Chinese local donkeys, Reference intervals, Hematological parameters, Serum biochemistry, Farm origin variation, Equine health monitoring

## Abstract

**Background:**

Reliable interpretation of hematological and serum biochemical test results in donkeys benefits from the use of population- and management-system-adapted reference intervals (RIs). However, standardized and CLSI-compliant RIs for Chinese local donkeys are still unavailable, limiting their clinical and epidemiological application. This study aimed to establish hematological and serum biochemical RIs for Chinese local donkeys using a large, multi-farm dataset, and to evaluate the effects of farm origin, age, and sex on these parameters. Blood samples were collected from healthy donkeys raised under different conservation levels and production systems, and RIs were constructed following Clinical and Laboratory Standards Institute (CLSI) C28-A3 guidelines using non-parametric methods.

**Results:**

A total of 1,163 serum samples collected from five geographically distinct donkey farms were included for serum biochemical analysis. The study population was sourced from national conservation farms comprising Dong’e and Wudi, provincial conservation farms comprising Yucheng and Zhongrun, and one local farm in Xinjiang. Twenty serum biochemical parameters covering protein metabolism, liver function, energy and lipid metabolism, mineral balance, and renal function were assessed. Hematological analyses were performed using samples obtained from 935 donkeys from the Dong’e farm. The results demonstrated that farm origin was the primary source of variation for most serum biochemical parameters. In contrast, although age- and sex-related differences were observed for certain variables, Harris and Boyd partitioning analysis indicated that these effects were not of sufficient magnitude to justify separate reference intervals for either biochemical or hematological parameters. Juvenile donkeys exhibited physiological trends consistent with growth and immune system maturation; however, these differences remained within the limits of non-partitioning criteria. Overall, the reference intervals established in this study differed from those reported in horses and donkeys from other regions, highlighting the substantial influence of population background and management conditions on physiological profiles.

**Conclusions:**

This study provides the first large-scale, CLSI-compliant hematological and serum biochemical reference intervals for Chinese local donkeys. The findings indicate that population- and management-system-adapted reference intervals are important for accurate clinical interpretation. These reference values offer a useful baseline for veterinary diagnosis, health monitoring, and conservation management of Chinese donkey populations.

**Supplementary Information:**

The online version contains supplementary material available at 10.1186/s12917-026-05556-y.

## Introduction

The donkey (*Equus asinus*) holds a unique position in livestock production, ecological agriculture, and local economies in China—not only as a traditional source of draft power and meat, but also increasingly for donkey milk production and conservation research [1]. Accurate interpretation of clinical hematological and serum biochemical data relies on species-specific reference intervals (RIs) [2]. Studies have demonstrated that reference values established for horses cannot be directly applied to donkeys [3], and that geographical region, breed, and management practices significantly influence hematological and biochemical parameters [2, 4]. Therefore, localized, methodologically consistent RIs tailored to the target population are essential to support clinical decision-making and research. Existing studies on Chinese donkeys mostly focus on mean comparisons and lack farm-specific, CLSI-compliant RIs, limiting their direct applicability for individual clinical diagnosis [5, 6].

Although international studies have established hematological and biochemical RIs for various donkey breeds and regions (e.g., the UK, Brazil, Portugal—Miranda donkeys—and recent multi-site studies in the USA), these works also emphasize that age, sex, analytical instruments/methods, and management conditions can affect results, thereby limiting comparability across regions [2, 7–10]. In China, fragmented biochemical and physiological studies exist for local donkey breeds (notably meat/milk donkeys such as the Dezhou donkey), but systematic, nationally or cross-provincially representative RIs are lacking [6, 11]. Moreover, existing local studies often focus on dietary interventions or specific physiological stages (e.g., lactation) rather than establishing standardized RIs for clinical interpretation [5, 6]. To address these gaps, the present study combines international best practices with representative sampling and strict quality control in Chinese local donkeys to fill this reference interval gap [8, 12].

Therefore, the present study was designed to systematically establish and evaluate reference intervals for commonly used hematological and serum biochemical parameters in Chinese local donkeys, while simultaneously quantifying the effects of sex, age, and geographical/management differences on these parameters. To achieve this objective, we collected blood samples from over 1,000 healthy donkeys across five geographically distinct farms representing different conservation levels and production systems. Reference intervals were established following strict CLSI C28-A3 guidelines [13], and comprehensive statistical analyses were performed to evaluate the influence of biological and environmental factors on blood parameters. The findings are expected to provide validated numerical baselines for clinical diagnosis, herd health monitoring, and breed conservation in Chinese donkey populations.

## Results

### Inter-system variation in serum biochemical parameters

Serum biochemical parameters, analysed after combining sexes, showed significant variation among donkey populations from different conservation farms based on group comparisons of central tendency (Table 1). Several parameters related to protein and nutritional status (ALB, TP), liver function and bilirubin metabolism (ALP, ALT, AST, GGT, DBIL, TBIL), energy and lipid metabolism (GLU, TCH, LIP), mineral metabolism (Ca, Mg, P, Fe), and renal function (CR, UA) differed significantly among farms (*P* < 0.05). It should be noted that Table 1 presents summary statistics for group comparisons rather than reference intervals. The actual reference intervals (2.5th–97.5th percentiles) for each conservation farm are provided in Supplementary Table S1.

Overall, provincial-level genetic resource conservation farms exhibited higher upper reference limits for several liver enzymes and serum calcium, whereas Xinjiang farms showed generally lower levels of ALP, GGT, LIP, bilirubin, and creatinine, indicating that serum biochemical baselines in donkeys are strongly farm-specific. These results suggest that farm origin should be considered a primary stratification factor when establishing serum biochemical reference intervals.

**Table 1:** Inter-population variation of donkey serum biochemical indexes in different conservation farms

Indicator	National Genetic Conservation Farm	Provincial Genetic Conservation Farm	Xinjiang Donkey Farm
Protein & nutritional status			
ALB(g/L)	26.14 (23.55–28.32)^a^	30.44 (27.03–32.49)^b^	23.73 (22.48–24.59)^c^
TP(g/L)	63.5 (48.8–71.9)^a^	43.8 (41.25–46.4)^b^	42.5 (40.5–44.95)^b^
Liver function & bilirubin metabolism			
ALP(U/L)	234.94 (177.86–301.51)^a^	282.62 (251.99–323.64)^b^	148.89 (123.15–168.7)^c^
ALT(U/L)	8.58 (7.03–10.22)^a^	8.97 (7.19–11.57)^b^	10.54 (9.2–12.11)^c^
AST(U/L)	301.68 (255.18–355.96)^a^	375.64 (337.06–439.4)^b^	260.96 (221.72–285.86)^c^
GGT(U/L)	46.91 (35.93–58.49)^a^	39.89 (30.15–50.89)^b^	16.74 (14.36–19.66)^c^
DBIL(µmol/L)	5.02 (4.02–6.72)^a^	6.11 (4.94–8.84)^b^	4.46 (3.49–5.93)^c^
TBIL(µmol/L)	4.94 (3.79–6.59)^a^	3.35 (2.67–4.84)^b^	4.15 (3.22–5.3)^c^
Digestive & pancreatic function			
AMY(U/L)	5.06 (3.36–7.04)^a^	7.61 (5.56–9.75)^b^	3.9 (2.58–5.43)^c^
Energy & lipid metabolism			
GLU(mmol/L)	3.51 (2.86–4.27)^a^	3.24 (2.82–3.8)^b^	3.18 (2.84–3.7)^b^
TG(mmol/L)	0.49 (0.34–0.71)^a^	0.4 (0.3–0.7)^b^	0.4 (0.3–0.6)^b^
TCH(mmol/L)	1.82 (1.55–2.1)^a^	1.55 (1.37–1.67)^b^	1.38 (1.24–1.49)^c^
LIP(U/L)	23 (20–27)^a^	19 (17–21)^b^	16 (15–18)^c^
Muscle & tissue integrity			
CK(U/L)	205 (158–268)^a^	228 (190–289.5)^b^	139 (112–161)^c^
Mineral & electrolyte metabolism			
Ca(mmol/L)	2.48 (2.34–2.65)^a^	2.85 (2.71–2.99)^b^	2.49 (2.4–2.58)^a^
Fe(µmol/L)	18 (15.1–21.6)^a^	20.9 (16.85–24.5)^b^	16.4 (13–19.6)^c^
Mg(mmol/L)	0.82 (0.71–0.92)^a^	0.6 (0.55–0.64)^b^	0.68 (0.62–0.74)^c^
P(mmol/L)	1.08 (0.85–1.35)^a^	1.06 (0.92–1.19)^a^	0.62 (0.48–0.75)^b^
Renal function & purine metabolism			
CR(µmol/L)	92.61 (80.49–106.31)^a^	82.69 (71.7–96.67)^b^	59.58 (49.98–69.69)^c^
UA(µmol/L)	31.26 (24.77–38.89)^a^	18.68 (15.77–22.86)^b^	29.47 (20.56–45.24)^a^

Values are presented as median (interquartile range, IQR). Superscript letters (a, b, c) indicate statistically significant differences in central tendency among farms (*P* < 0.05), based on group comparisons, and do not represent differences in reference interval limits. Biochemical indicators are grouped according to their main physiological functions. Abbreviations: ALB, albumin; TP, total protein; ALP, alkaline phosphatase; ALT, alanine aminotransferase; AST, aspartate aminotransferase; GGT, γ-glutamyl transferase; DBIL, direct bilirubin; TBIL, total bilirubin; AMY, amylase; GLU, glucose; TG, triglycerides; TCH, total cholesterol; CK, creatine kinase; Ca, calcium; Fe, iron; Mg, magnesium; P, phosphorus; CR, creatinine; UA, uric acid; LIP, lipase.

### Within-farm sex differences in serum biochemistry

Sex-related differences in serum biochemical parameters varied across farms (Table 2). At the national genetic conservation farms, multiple parameters differed significantly between sexes, primarily involving liver function and lipid metabolism (*P* < 0.001). At provincial conservation farms, only total bilirubin (TBIL) showed a significant sex difference (*P* < 0.01). At the Xinjiang donkey farm, several parameters showed statistically significant sex differences, including albumin (ALB), amylase (AMY), aspartate aminotransferase (AST), and total protein (TP) (*P* < 0.01 or *P* < 0.001). However, Harris and Boyd’s statistical approach indicated that sex-related differences were not sufficient to justify partitioning reference intervals (Supplementary Table S2), suggesting that sex-specific reference intervals were not required for these biochemical parameters across farms.

**Table 2:** Comparison of serum biochemical parameters between male and female adult donkeys across different farms

Indicator	National Genetic Conservation Farm			Provincial Genetic Conservation Farm			Xinjiang Donkey Farm		
	Male (Mean ± SD)	Female (Mean ± SD)	Sig.	Male (Mean ± SD)	Female (Mean ± SD)	Sig.	Male (Mean ± SD)	Female (Mean ± SD)	Sig.
Protein & nutritional status									
ALB(g/L)	25.45 ± 2.86	26.90 ± 3.56	***	29.18 ± 3.55	29.67 ± 4.30	ns	25.66 ± 1.76	23.53 ± 1.88	**
TP(g/L)	56.19 ± 13.43	59.56 ± 13.79	ns	45.48 ± 4.34	44.03 ± 4.97	ns	47.09 ± 3.11	42.41 ± 3.27	***
Liver function & bilirubin metabolism									
ALP(U/L)	265.23 ± 72.69	204.86 ± 68.33	***	295.69 ± 74.19	290.47 ± 68.26	ns	158.37 ± 37.20	148.79 ± 41.47	ns
ALT(U/L)	6.21 ± 2.91	9.21 ± 3.68	***	9.55 ± 2.69	9.72 ± 3.54	ns	10.63 ± 2.45	11.03 ± 2.81	ns
AST(U/L)	286.85 ± 65.56	323.59 ± 74.33	***	378.07 ± 93.08	392.14 ± 92.70	ns	205.12 ± 65.80	263.96 ± 50.57	**
GGT(U/L)	37.07 ± 13.60	48.42 ± 15.22	***	46.55 ± 14.76	38.53 ± 14.31	ns	21.90 ± 6.72	17.27 ± 5.17	ns
DBIL(µmol/L)	4.87 ± 1.67	6.71 ± 5.99	***	7.93 ± 3.09	6.97 ± 2.90	ns	7.03 ± 4.21	4.88 ± 1.91	ns
TBIL(µmol/L)	3.50 ± 1.50	6.08 ± 5.61	***	4.76 ± 1.82	3.57 ± 1.48	**	5.93 ± 3.17	4.35 ± 1.50	ns
Digestive & pancreatic function									
AMY(U/L)	5.09 ± 6.90	5.83 ± 3.09	ns	10.04 ± 11.33	7.94 ± 4.22	ns	7.61 ± 3.25	4.14 ± 2.63	**
Energy & lipid metabolism									
GLU(mmol/L)	3.27 ± 0.84	3.71 ± 4.70	ns	3.47 ± 0.68	3.24 ± 0.74	ns	3.90 ± 0.62	3.27 ± 0.77	ns
TG(mmol/L)	0.40 ± 0.19	0.70 ± 0.72	***	0.57 ± 0.27	0.47 ± 0.26	ns	0.56 ± 0.28	0.45 ± 0.22	ns
TCH(mmol/L)	1.52 ± 0.29	1.87 ± 0.50	***	1.53 ± 0.19	1.53 ± 0.26	ns	1.61 ± 0.34	1.37 ± 0.22	ns
LIP(U/L)	20.10 ± 3.19	23.68 ± 6.28	***	22.53 ± 9.71	19.18 ± 3.59	ns	18.18 ± 2.32	17.02 ± 4.13	ns
Muscle & tissue integrity									
CK(U/L)	201.64 ± 92.15	221.67 ± 84.86	ns	249.56 ± 150.99	257.37 ± 101.06	ns	110.64 ± 30.54	145.50 ± 43.92	ns
Mineral & electrolyte metabolism									
Ca(mmol/L)	2.53 ± 0.27	2.52 ± 0.25	ns	2.91 ± 0.30	2.84 ± 0.25	ns	2.52 ± 0.14	2.49 ± 0.15	ns
Fe(µmol/L)	17.35 ± 5.39	19.11 ± 5.27	ns	18.83 ± 6.22	21.66 ± 5.38	ns	17.50 ± 5.15	16.70 ± 5.57	ns
Mg(mmol/L)	0.66 ± 0.12	0.86 ± 0.14	***	0.58 ± 0.07	0.61 ± 0.08	ns	0.64 ± 0.08	0.68 ± 0.09	ns
P(mmol/L)	1.09 ± 0.44	1.02 ± 0.44	ns	1.14 ± 0.24	1.06 ± 0.44	ns	0.77 ± 0.37	0.63 ± 0.42	ns
Renal function & purine metabolism									
CR(µmol/L)	98.59 ± 17.42	96.03 ± 20.84	ns	87.94 ± 20.65	85.76 ± 19.68	ns	79.81 ± 28.91	61.04 ± 15.99	ns
UA(µmol/L)	29.50 ± 17.46	34.26 ± 15.37	ns	19.22 ± 7.23	20.49 ± 7.02	ns	52.17 ± 36.15	34.99 ± 20.31	ns

Data are presented as mean ± standard deviation (Mean ± SD). Significance levels: ns, *P* ≥ 0.05; * *P* < 0.05; ** *P* < 0.01; *** *P* < 0.001. Biochemical indicators are grouped according to their main physiological functions. Adult donkeys were defined as ≥ 18 months of age

### Age-related differences in serum biochemical parameters at the Dong’e donkey farm

At the Dong’e Donkey Farm, one of the National Genetic Conservation Farm, juvenile donkeys showed significantly higher levels of ALP, ALT, GGT, LIP, P, TCH, and TP than adult donkeys, whereas adults exhibited higher concentrations of ALB, Ca, and CR (Table 3; *P* < 0.05 or *P* < 0.001). No significant age-related differences were detected for AST, AMY, GLU, TG, TBIL, or UA.

However, application of the Harris and Boyd method indicated that the observed age-related differences were not sufficient to justify partitioning of reference intervals. Accordingly, age-specific stratification was not required, and sex-combined, farm-specific reference intervals were ultimately adopted in the present study (Supplementary Table S1).

**Table 3:** Comparison of serum biochemical indicators between adult and juvenile donkeys in the Dong ‘e donkey farm

Dong ‘e donkey farm			
Indicator	Adult Mean ± SD	Juvenile Mean ± SD	Sig.
Protein & nutritional status			
ALB(g/L)	26.53 ± 3.45	25.66 ± 3.82	***
TP(g/L)	58.7 ± 13.76	62.98 ± 12.62	***
Liver function & bilirubin metabolism			
ALP (U/L)	220.23 ± 74.21	270.86 ± 95.95	***
ALT (U/L)	8.44 ± 3.73	9.45 ± 4.9	***
AST (U/L)	314.24 ± 73.88	311.67 ± 97.83	ns
GGT (U/L)	45.53 ± 15.62	49.15 ± 14.86	***
DBIL (µmol/L)	6.24 ± 5.3	5.91 ± 3.06	ns
TBIL (µmol/L)	5.42 ± 5.03	5.9 ± 2.53	ns
Digestive & pancreatic function			
AMY (U/L)	5.64 ± 4.38	5.9 ± 5.91	ns
Energy & lipid metabolism			
GLU (mmol/L)	3.6 ± 4.08	3.82 ± 1.08	ns
TG (mmol/L)	0.62 ± 0.64	0.58 ± 0.3	ns
TCH (mmol/L)	1.78 ± 0.48	1.95 ± 0.43	***
LIP (U/L)	22.77 ± 5.86	23.87 ± 5.25	**
Muscle & tissue integrity			
CK(U/L)	216.57 ± 87.1	231.88 ± 106.52	*
Mineral & electrolyte metabolism			
Ca(mmol/L)	2.53 ± 0.26	2.48 ± 0.25	*
Fe(µmol/L)	18.66 ± 5.35	19.16 ± 6.94	ns
Mg(mmol/L)	0.81 ± 0.16	0.82 ± 0.14	ns
P(mmol/L)	1.04 ± 0.44	1.23 ± 0.48	***
Renal function & purine metabolism			
CR (µmol/L)	96.68 ± 20.04	91.7 ± 18.1	***
UA (µmol/L)	33.05 ± 16.04	34.16 ± 12.16	ns

Values are presented as mean ± SD. Adult donkeys were defined as ≥ 18 months of age and juvenile donkeys as < 18 months of age, based on physiological maturity criteria. Statistical significance was assessed using one-way ANOVA or Welch ANOVA where appropriate. Significance levels are indicated as *P* < 0.05 (*), *P* < 0.01 (**), and *P* < 0.001 (***); ns indicates not significant

### Age-associated differences in hematological parameters

Compared with adult donkeys, juveniles exhibited significantly higher total white blood cell counts (WBC) and platelet counts (PLT), as well as higher plateletcrit (PCT) and red cell distribution width (RDW-CV). For white blood cell subpopulations, juveniles showed a significantly higher neutrophil percentage [Neu (%)] and lower lymphocyte percentage [Lym (%)], monocyte percentage [Mon (%)], eosinophil percentage [Eos (%)], and basophil percentage [Bas (%)] compared with adults. However, the absolute counts of lymphocytes, monocytes, eosinophils, and basophils did not differ significantly between age groups, indicating that the observed differences primarily reflect a redistribution of leukocyte subpopulations rather than changes in total cell numbers (Table 4; *P* < 0.05 or *P* < 0.001).

In the erythrocyte system, juvenile donkeys had higher red blood cell counts (RBC) but significantly lower mean corpuscular volume (MCV), mean corpuscular hemoglobin (MCH), and mean corpuscular hemoglobin concentration (MCHC) compared with adults. In contrast, adult donkeys showed significantly higher MCV, MCH, MCHC, and RDW-SD. No significant differences were observed between age groups in hemoglobin concentration (HGB) or hematocrit (HCT).

**Table 4:** Age-related differences in hematological parameters

Indicator	Dong ‘e donkey farm		
	Adult Mean ± SD	Juvenile Mean ± SD	Sig.
White cell line			
WBC (10^9/L)	13.22 ± 3.54	15.33 ± 19.24	*
Neu (10^9/L)	5.66 ± 1.94	7.34 ± 15.14	*
Neu (%)	43.02 ± 9.23	45.89 ± 10.37	***
Lym (10^9/L)	6.33 ± 2.33	6.50 ± 2.27	ns
Lym (%)	47.52 ± 8.97	44.73 ± 9.56	***
Mon (10^9/L)	0.51 ± 0.35	0.77 ± 3.11	ns
Mon (%)	3.84 ± 2.42	4.42 ± 2.37	***
Eos (10^9/L)	0.72 ± 0.32	0.72 ± 0.58	ns
Eos (%)	5.60 ± 2.37	4.94 ± 2.74	***
Bas (10^9/L)	0.00 ± 0.01	0.00 ± 0.03	ns
Bas (%)	0.02 ± 0.05	0.01 ± 0.04	***
Red cell line			
RBC (10^12/L)	7.04 ± 1.24	7.85 ± 1.45	***
HCT (%)	40.81 ± 6.58	41.40 ± 7.35	ns
HGB (g/L)	130.09 ± 20.82	130.01 ± 23.43	ns
MCH (pg)	18.59 ± 1.44	16.68 ± 1.89	***
MCHC (g/L)	319.05 ± 12.17	314.43 ± 14.42	***
MCV (fL)	58.27 ± 4.07	53.07 ± 5.63	***
RDW-CV (%)	21.80 ± 1.36	22.70 ± 1.61	***
RDW-SD (fL)	43.66 ± 2.15	41.65 ± 3.32	***
Platelets			
MPV (fL)	6.72 ± 1.19	6.54 ± 1.14	*
PCT (%)	0.16 ± 0.05	0.20 ± 0.08	***
PDW (%)	15.74 ± 0.51	15.49 ± 0.46	***
PLT (10^9/L)	248.67 ± 88.53	314.35 ± 128.39	***

Data are presented as mean ± standard deviation (SD). Adult donkeys were defined as ≥ 18 months of age and juvenile donkeys as < 18 months of age. Differences between adult and juvenile donkeys were assessed using one-way ANOVA or Welch ANOVA, depending on data normality and homogeneity of variance. Sig. indicates statistical significance: * *P* < 0.05, ** *P* < 0.01, *** *P* < 0.001; ns, not significant

### Sex-related differences in hematological parameters

Multiple hematological parameters exhibited significant sex-related differences in adult donkeys (Table 5). Adult females showed significantly higher hematocrit (HCT), hemoglobin concentration (HGB), mean corpuscular hemoglobin (MCH), mean corpuscular volume (MCV), as well as platelet volume–related indices (MPV and PDW) and RDW-SD compared with adult males. In contrast, adult males had significantly higher red blood cell counts (RBC), platelet counts (PLT), plateletcrit (PCT), RDW-CV, total white blood cell counts (WBC), and lymphocyte counts and proportions than adult females (*P* < 0.05 or *P* < 0.001).

**Table 5:** Sex-related differences in hematological parameters

Indicator	Dong ‘e donkey farm		
	Male Mean ± SD	Female Mean ± SD	Sig.
White cell line			
WBC (10^9/L)	13.97 ± 3.23	13.01 ± 3.60	*
Neu (10^9/L)	5.80 ± 1.86	5.61 ± 1.96	ns
Neu (%)	41.76 ± 9.20	43.37 ± 9.22	ns
Lym (10^9/L)	6.95 ± 2.29	6.16 ± 2.32	**
Lym (%)	49.39 ± 9.44	46.99 ± 8.78	*
Mon (10^9/L)	0.47 ± 0.17	0.52 ± 0.38	ns
Mon (%)	3.34 ± 0.86	3.98 ± 2.69	***
Eos (10^9/L)	0.74 ± 0.28	0.71 ± 0.33	ns
Eos (%)	5.47 ± 1.95	5.63 ± 2.48	ns
Bas (10^9/L)	0.01 ± 0.01	0.00 ± 0.01	***
Bas (%)	0.05 ± 0.07	0.02 ± 0.04	***
Red cell line			
RBC (10^12/L)	7.42 ± 1.48	6.93 ± 1.14	**
HCT (%)	39.40 ± 7.88	41.21 ± 6.11	*
HGB (g/L)	125.82 ± 26.23	131.30 ± 18.89	*
MCH (pg)	16.97 ± 0.85	19.04 ± 1.22	***
MCHC (g/L)	319.34 ± 9.32	318.97 ± 12.88	ns
MCV (fL)	53.15 ± 2.40	59.72 ± 3.17	***
RDW-CV (%)	23.21 ± 1.18	21.41 ± 1.12	***
RDW-SD (fL)	42.64 ± 2.10	43.95 ± 2.08	***
Platelets			
MPV (fL)	6.11 ± 0.85	6.89 ± 1.21	***
PCT (%)	0.18 ± 0.04	0.16 ± 0.05	***
PDW (%)	15.42 ± 0.34	15.83 ± 0.51	***
PLT (10^9/L)	307.27 ± 84.41	232.09 ± 82.53	***

Values are presented as mean ± SD. Differences between male and female donkeys were assessed using one-way ANOVA or Welch ANOVA, depending on data normality and homogeneity of variance. ns, not significant. Significance levels: *P* < 0.05 (*), *P* < 0.01 (**), *P* < 0.001 (***)

### Establishment of reference intervals for hematological parameters

Outliers were removed using Tukey’s method. Reference intervals (RIs) for donkeys in the Dong’e donkey farm were then established based on 700–929 samples (Table 6). Most parameters, particularly those related to red blood cells, showed symmetric distributions with relatively narrow intervals, whereas white blood cell subpopulations and platelet-related parameters exhibited wider intervals, reflecting higher biological variability. Basophil counts were near zero, while eosinophils, lymphocytes, and neutrophils comprised the primary white blood cell populations. Supplementary Table S1 provides detailed reference intervals for all hematological parameters measured in this farm. Most observed differences fell within overlapping ranges, supporting the use of unified reference intervals for routine clinical applications. The Harris and Boyd method further confirmed that neither age- nor sex-based partitioning was necessary for these hematological parameters (Supplementary Table S2). These reference intervals provide a reliable baseline for health assessment and comparative studies in donkey populations. However, as all hematological samples were obtained from a single farm, validation in donkeys from other geographical regions and management systems is recommended before broader application.

**Table 6:** Reference intervals for hematological indicators

Indicator	*n*	Mean	SD	Median	Min	Max	Reference intervals
White cell line							
WBC (10^9/L)	898	13.7	3.17	13.6	5.11	22.41	7.7-20.43
Neu (10^9/L)	875	5.8	1.62	5.55	1.39	10.42	2.96–9.50
Neu (%)	893	43.79	8.29	43.4	21.6	66.3	28.03–61.3
Lym (10^9/L)	907	6.29	1.93	6.15	0.96	11.34	2.58–10.34
Lym (%)	899	46.55	8.11	47	25.2	67.8	30.45–62.46
Mon (10^9/L)	871	0.51	0.2	0.48	0.04	1.13	0.18–0.98
Mon (%)	868	3.66	1.18	3.5	0.8	7.1	1.8–6.4
Eos (10^9/L)	899	0.67	0.31	0.64	0.05	1.51	0.13–1.34
Eos (%)	911	5.06	2.3	4.9	0.3	11.5	1.1-10.03
Bas (10^9/L)	700	0	0	0	0	0	0
Bas (%)	794	0	0	0	0	0	0
Red cell line							
RBC (10^12/L)	903	7.4	1.13	7.36	4.38	10.48	5.37–9.75
HCT (%)	899	40.68	5.3	40.3	27.7	54.8	30.6–51.6
HGB(g/L)	905	128.85	16.58	128	89	174	96.6–164
MCH (pg)	928	17.55	1.92	17.6	12	23.1	13.72–20.9
MCHC (g/L)	904	317.59	11.13	319	287	346	293.58–337
MCV (fL)	929	55.42	5.59	55.9	39	72	44.22–64.88
RDW-CV (%)	906	22.19	1.31	22.1	18.6	25.7	19.76–24.94
RDW-SD (fL)	915	42.49	2.68	42.6	35.4	49.4	37.2-47.72
Platelets							
MPV (fL)	925	6.6	1.14	6.4	4.1	9.9	5.01–9.1
PCT (%)	899	0.18	0.06	0.18	0.03	0.33	0.07–0.29
PDW (%)	916	15.58	0.45	15.5	14.4	16.9	14.9-16.51
PLT (10^9/L)	920	280.22	107.36	275	12	592	78.98–501

Reference intervals (RIs) were calculated using the non-parametric 2.5th–97.5th percentile method according to CLSI C28-A3 guidelines. All hematological samples were collected from a single farm (Dong’e County, Shandong Province)

## Discussion

The present study represents the largest systematic effort to establish CLSI-compliant reference intervals for Chinese local donkeys, encompassing over 1,000 individuals across geographically and managerially distinct farm systems. The results demonstrated that farm origin was the primary factor driving variability in serum biochemical profiles, a finding with important implications for clinical interpretation and diagnostic decision-making. Significant differences among farms were observed across multiple physiological systems, including protein and nutritional status, hepatic function and bilirubin metabolism, energy and lipid metabolism, mineral homeostasis, and renal function. These patterns indicate that feeding systems, management intensity, and genetic background are the main contributors to variation in serum biochemical indicators [14]. The observed inter-farm heterogeneity can be attributed to several mechanistic factors. Donkeys from the national genetic conservation farm, maintained under unified feeding regimens and highly centralized management, exhibited overall stable reference intervals. In contrast, donkeys from Xinjiang farms, characterized predominantly by grazing-based systems and crossbred populations, exhibited generally lower levels or narrower reference intervals for multiple liver enzyme, lipid, and energy metabolism indicators. These patterns may be associated with higher physical activity levels [15, 16], differences in dietary composition [17, 18], and greater genetic background diversity [19, 20], consistent with previous findings in donkeys and other equids raised under different husbandry systems [2, 4]. Provincial conservation farms operating under semi-standardized production systems showed reduced variability in several metabolism- and liver function–related indicators, highlighting the positive effect of management standardization in mitigating physiological fluctuations [21, 22].

Building upon these farm-level observations, total protein (TP) and albumin (ALB), as key indicators of protein metabolism, are important biomarkers of nutritional status in animals [23]. Previous studies have shown that under stable intensive housing conditions, serum TP levels are higher in male and juvenile donkeys, whereas ALB concentrations tend to be higher in adult or female donkeys [9, 24]. However, in the present study, some female donkeys from the Xinjiang farm exhibited lower ALB levels than males. This discrepancy may be attributed to sex-related physiological regulation. Sex hormones play an important role in hepatic protein synthesis; estrogen may suppress albumin production under certain conditions, whereas androgens promote protein anabolism [25, 26]. Population-based studies have also shown that serum albumin levels in females tend to be lower or decline more rapidly [27]. In addition, differences in nutrient allocation, environmental stress, and physiological states such as lactation may further contribute to reduced ALB levels in females [28–30].

Closely related to protein metabolism, hepatic function indicators, including ALT, AST, and GGT, were higher in adult female and juvenile donkeys, a pattern consistent with the observations reported by Ihsan Kısadere [24]. In contrast, adult male donkeys from the national conservation farm exhibited significantly higher ALP levels than females, which may be associated with restricted physical activity under individual stall housing and the stimulatory effects of androgens on bone metabolism [31, 32]. The higher ALP levels in confined males may also reflect enhanced osteoblastic activity secondary to testosterone-mediated bone remodeling, as ALP serves as a marker of both hepatic and skeletal metabolism. Furthermore, regarding bilirubin metabolism, elevated TBIL and DBIL levels in females from the national conservation farm may be related to sex hormone–regulated differences in UGT-mediated bilirubin metabolism [33]. Conversely, higher TBIL levels were observed in males at the provincial farm, suggesting that sex-related differences in bilirubin concentrations may be modulated by environmental conditions and hepatic clearance efficiency under different management systems [34, 35]. Age did not exert a significant effect on these bilirubin indicators [36].

In addition to hepatic parameters, serum glucose and lipid profiles provide valuable insight into metabolic adaptation under different management systems. In the present study, serum glucose (GLU) levels were comparable to those reported for Martina Franca donkeys [12], Kyrgyz donkeys [24], and Brazilian donkeys [30]. Serum triglyceride (TG) concentrations were higher than those reported for Martina Franca and Kyrgyz donkeys [12, 24], but lower than those observed in Pêga donkeys [28] and American donkeys [2], suggesting that inter-population variation may be associated with differences in dietary energy density, feeding frequency, and metabolic adaptation to local environments. Notably, the higher TG levels observed in female donkeys may be related to sex hormone–mediated regulation of key enzymes involved in triglyceride synthesis and oxidation, as well as sex-specific control of hepatic very-low-density lipoprotein triglyceride (VLDL-TG) production and lipid storage [37].

Furthermore, the age-related biochemical differences observed in this study may reflect the coordinated maturation of hepatic and systemic metabolic systems rather than alterations in a single metabolic pathway [38, 39]. In mammals, the liver undergoes continuous postnatal development, during which metabolic enzyme systems (such as cytochrome P450), transport proteins, and synthetic functions progressively mature, leading to systematic differences in biochemical profiles across growth stages [40, 41]. Dynamic changes in hepatic metabolic capacity during development can directly influence serum enzymatic indices [42]. In this study, the higher levels of ALT, ALP, and GGT observed in young donkeys may be associated with ongoing hepatic maturation and tissue remodeling [42]. From a physiological perspective, juvenile donkeys are expected to show relatively higher activities of enzymes linked to growth and tissue turnover because hepatic parenchyma, biliary transport, and detoxification systems are still developing; therefore, mild increases in ALT, ALP, and GGT can be interpreted as age-related maturation rather than pathological hepatocellular injury, particularly when values remain within the established RI [43].

In addition, lipid and energy metabolism exhibit pronounced age-dependent patterns. Total cholesterol (TCH) concentrations were significantly higher in juvenile donkeys than in adults, which is consistent with increased cholesterol demand and enhanced hepatic synthetic activity during growth [44]. Meanwhile, elevated serum lipase (LIP) activity in juveniles likely reflects increased metabolic demands during rapid growth [45]. Enhanced lipid mobilization and turnover are essential for supporting tissue development and are more dynamically regulated during early life stages [46–48]. In contrast, metabolic activity becomes more stable with age, which may contribute to relatively lower LIP activity in adult individuals [49]. These age-associated differences may also reflect the fact that growing animals require greater membrane lipid synthesis, steroid precursor availability, and energy turnover to support skeletal and soft tissue expansion, whereas adults prioritize homeostatic maintenance [50, 51].

Extending the analysis to muscle integrity and renal function, creatine kinase (CK) did not differ significantly between sexes; however, Caldin reported an age-related high–low–high pattern in CK activity [36], which may be attributable to rapid muscle growth and exercise-induced microdamage during the juvenile stage, physiological stability in adulthood, and degenerative muscular changes in aged donkeys [52–54]. Serum creatinine (CR) increased with age, consistent with findings in Ragusano and Pêga donkeys [28, 33], reflecting increased creatinine production associated with greater total muscle mass [55]. Similarly, serum amylase (AMY) concentrations were higher in males than in females, which may reflect sex hormone–mediated regulation of amylase expression and androgen-associated enhancement of enzyme synthesis [56]. Although AMY values in the present study were comparable to those reported for British donkeys [7], discrepancies with some previous studies may be attributable to differences in laboratory methodologies, reagent batches, and analytical instrumentation [30]. When compared with horses and other donkey breeds, these renal and muscle-related baselines further underscore that values derived from equine reference populations should not be directly transferred to Chinese local donkeys without population-specific verification.

With respect to mineral and electrolyte homeostasis, serum calcium concentrations were significantly higher in adult donkeys than in juveniles, whereas phosphorus levels were higher in juvenile donkeys. This reciprocal pattern is consistent with the physiological shift from active skeletal growth to mineral homeostasis [57]. During early life, rapid bone formation requires substantial phosphorus for hydroxyapatite deposition, while calcium is increasingly retained and tightly regulated in adults to maintain extracellular homeostasis and neuromuscular function [58, 59]. Previous studies have demonstrated that calcium and phosphorus metabolism are developmentally regulated through coordinated endocrine control involving parathyroid hormone, vitamin D, and fibroblast growth factor 23 (FGF23), which together modulate intestinal absorption, renal reabsorption, and skeletal turnover across growth stages [60]. Moreover, among adult donkeys, females exhibited significantly higher serum magnesium concentrations than males, which may be related to estrogen-mediated regulation of magnesium absorption and homeostasis, consistent with established patterns of mineral metabolism in mammals [61]. Iron-related parameters did not show significant variation with age or sex. Serum uric acid (UA) concentrations did not differ significantly across age or sex groups, indicating that purine metabolism and renal excretion are maintained in a stable equilibrium in healthy donkey populations, consistent with findings reported in Pêga donkeys [31]. The calcium–phosphorus pattern observed here is physiologically meaningful for field practice because juveniles with low phosphorus or atypical calcium values may warrant closer nutritional review, while adults with unexpected mineral deviations should prompt evaluation of diet quality, grazing access, and possible subclinical metabolic disturbance [62].

Turning to the hematological findings, systematic variation attributable to both developmental stage and sex was observed, providing mechanistic insight into hematopoietic regulation in donkeys. In this study, juvenile donkeys exhibited significantly higher total white blood cell counts (WBC) and absolute neutrophil counts (Neu) compared with adults, accompanied by an increased neutrophil proportion [Neu (%)] and decreased lymphocyte proportion [Lym (%)]. This pattern suggests a constitutive redistribution among leukocyte subpopulations rather than an overall increase in immune cell numbers, reflecting the relative immaturity of the immune system and the heightened activity of innate immune components in juvenile individuals [63]. Concurrently, in the erythrocyte system, juveniles had higher red blood cell counts (RBC), but significantly lower mean corpuscular volume (MCV), mean corpuscular hemoglobin (MCH), and mean corpuscular hemoglobin concentration (MCHC), together with changes in red cell distribution width (RDW-CV, RDW-SD). These findings are broadly consistent with reports in Martina Franca and Catalonian donkeys [12, 64], reflecting active hematopoiesis and increased erythrocyte heterogeneity during growth [65]. Despite significant differences in erythrocyte morphology, hemoglobin concentration (HGB) and hematocrit (HCT) remained relatively stable across age groups, indicating that overall oxygen-carrying capacity is maintained through adjustments in cell number and volume [66]. Additionally, juvenile donkeys showed significantly higher platelet counts (PLT) and plateletcrit (PCT), but lower mean platelet volume (MPV) and platelet distribution width (PDW), a trend that differs from previous studies [12, 36]. This discrepancy may be attributed to the rapid growth phase of juveniles, during which active bone marrow hematopoiesis favors increased platelet production with smaller, more uniform platelets, resulting in elevated PLT and PCT alongside reduced MPV and PDW [67, 68]. Such changes likely represent physiologic, growth-related hematopoietic characteristics rather than platelet activation or pathological alterations. These age-related hematological patterns are consistent with ongoing hematopoietic maturation: erythrocyte indices evolve as oxygen transport capacity and red cell size stabilize, while platelet indices reflect marrow output and developmental platelet turnover [69, 70].

Parallel to the age-related differences, sex-related variations in hematological parameters reflect hormonal influences on hematopoiesis and immune function. Male donkeys exhibited higher total white blood cell counts (WBC) and absolute lymphocyte counts (Lym) compared with females, whereas females showed a relatively higher monocyte proportion [Mon (%)], suggesting that sex may influence immune cell distribution through hormonal or stress-related mechanisms [71, 72]. With regard to erythrocyte parameters, clear sexual dimorphism was evident: males had higher red blood cell counts (RBC) but lower mean corpuscular volume (MCV) and mean corpuscular hemoglobin (MCH) along with increased RDW-CV, whereas females showed higher hemoglobin concentration (HGB), hematocrit (HCT), and MCV, indicating larger erythrocyte size and greater oxygen-carrying capacity per cell [73, 74]. This pattern is consistent with the well-established role of androgens in stimulating erythropoiesis, primarily through upregulation of erythropoietin production and enhanced bone marrow activity, leading to increased RBC counts in males [75, 76]. Conversely, estrogens have been reported to exert modulatory or even inhibitory effects on erythropoiesis, which may contribute to lower RBC counts but relatively larger erythrocyte volume in females [77]. The elevated RDW-CV observed in males may further reflect increased heterogeneity in erythrocyte size, potentially associated with more active erythrocyte turnover or differential maturation dynamics under androgen influence [78–80]. This pattern of compensatory erythrocyte morphology in females may represent an adaptive mechanism to maintain oxygen delivery capacity despite lower cell counts. Correspondingly, in the platelet system, males exhibited higher platelet counts (PLT) and plateletcrit (PCT), while females showed higher mean platelet volume (MPV) and platelet distribution width (PDW), a pattern similar to that reported by da Silva et al., reflecting physiological regulation differences between platelet number and size [81]. Such sex-related differences in platelet indices are thought to reflect distinct regulatory mechanisms between platelet production and activation, with androgens generally promoting thrombopoiesis and platelet number, whereas estrogens are associated with enhanced platelet activation and larger platelet size [76, 82]. Higher MPV and PDW in females may indicate increased platelet reactivity and turnover, which has been reported in several mammalian species and linked to hormonal modulation of megakaryocyte maturation and platelet release [83–85]. In practical terms, these sex-associated hematological shifts are modest but relevant when interpreting borderline RBC or platelet results in healthy donkeys, especially in breeding animals or individuals under hormonal and nutritional fluctuation.

**Fig. 1:**
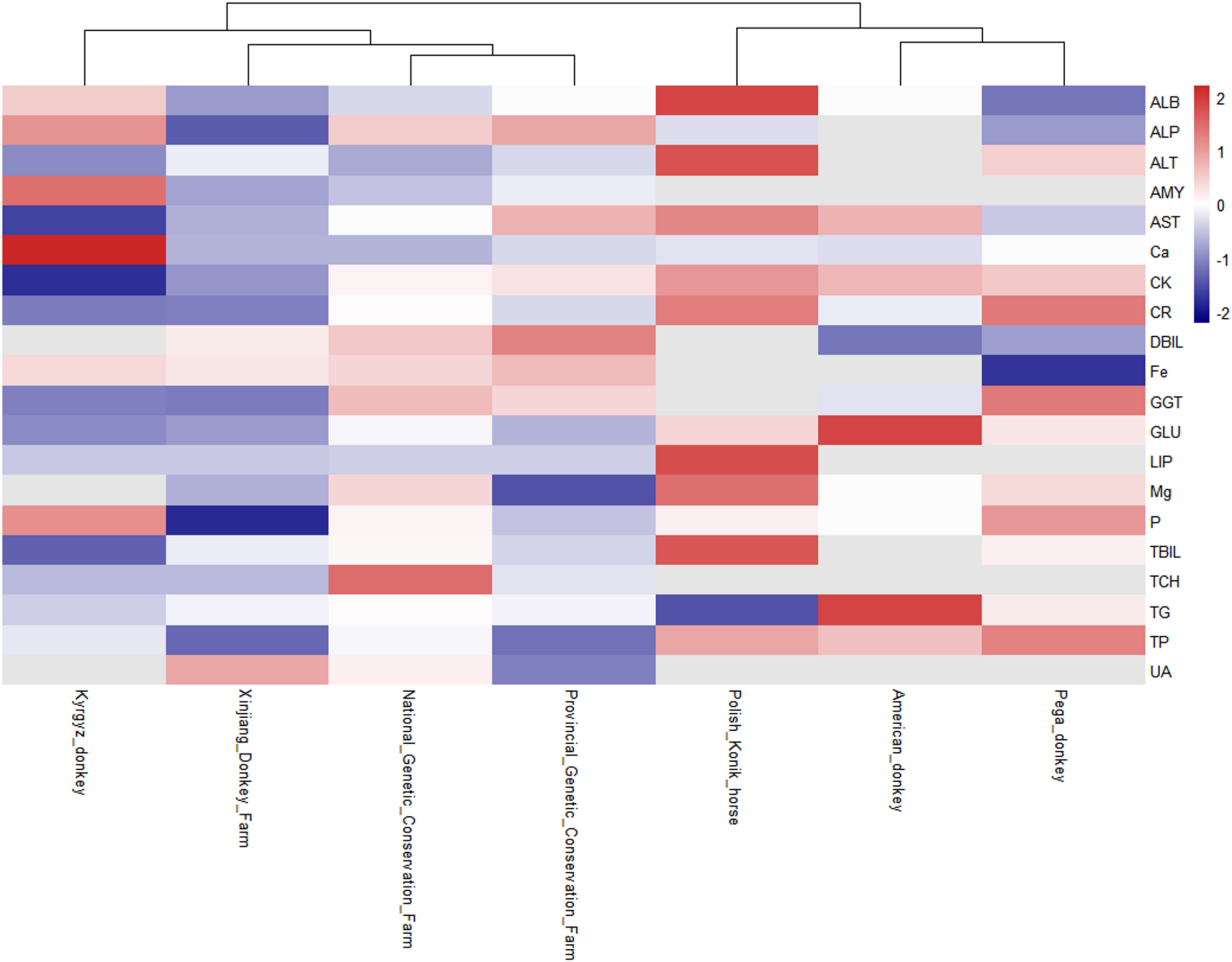
Cross-species comparison of reference intervals for serum biochemical parameters

Based on the hierarchical clustering results from the heatmap (Fig. 1), the reference intervals established from the National Genetic Conservation Farm, Provincial Genetic Conservation Farm, and Xinjiang Donkey Farm exhibited a generally consistent intermediate distribution across most serum biochemical parameters. Cluster analysis revealed that the two conservation farm populations first formed a closely related and relatively independent subcluster, whereas the Xinjiang Donkey Farm grouped with the Kyrgyz donkey into another branch. Together, these formed a major donkey clade on the left side of the dendrogram; however, they did not further merge into a completely unified cluster. Compared with this reference system, the Kyrgyz donkey showed a relatively high degree of similarity in the overall serum biochemical profile, suggesting physiological proximity to the established reference ranges, although noticeable deviations were observed in several key parameters, particularly Ca and AST. In contrast, the Polish Konik horse exhibited the most pronounced divergence from the reference intervals, mainly characterized by higher or distinctly different distribution patterns in DBIL and TCH. Furthermore, the American donkey and Pêga donkey showed clear separation from the reference group in parameters such as Fe, TG, and TP, indicating substantially different physiological patterns. These differences likely reflect considerable heterogeneity associated with geographic origin and breed background [12]. Overall, our RIs aligned more closely with some reported donkey populations than with horses, reinforcing that equid species and even donkey breeds differ substantially in baseline hematology and biochemistry [2]. Relative to horse-derived values, the present donkey intervals better capture the lower or differently distributed enzyme, lipid, and mineral patterns typical of donkeys, which are known to be more metabolically conservative and often less prone to the same reference shifts seen in horses [4].

The data were obtained from [2, 24, 28, 86]. Serum biochemical reference intervals of equids were converted to midpoints and standardized (z-score). Samples were clustered using Euclidean distance, with clustering applied only to columns. Missing values were shown as NA (grey). The heatmap was generated in R (v4.4.1) using heatmap.

**Fig. 2:**
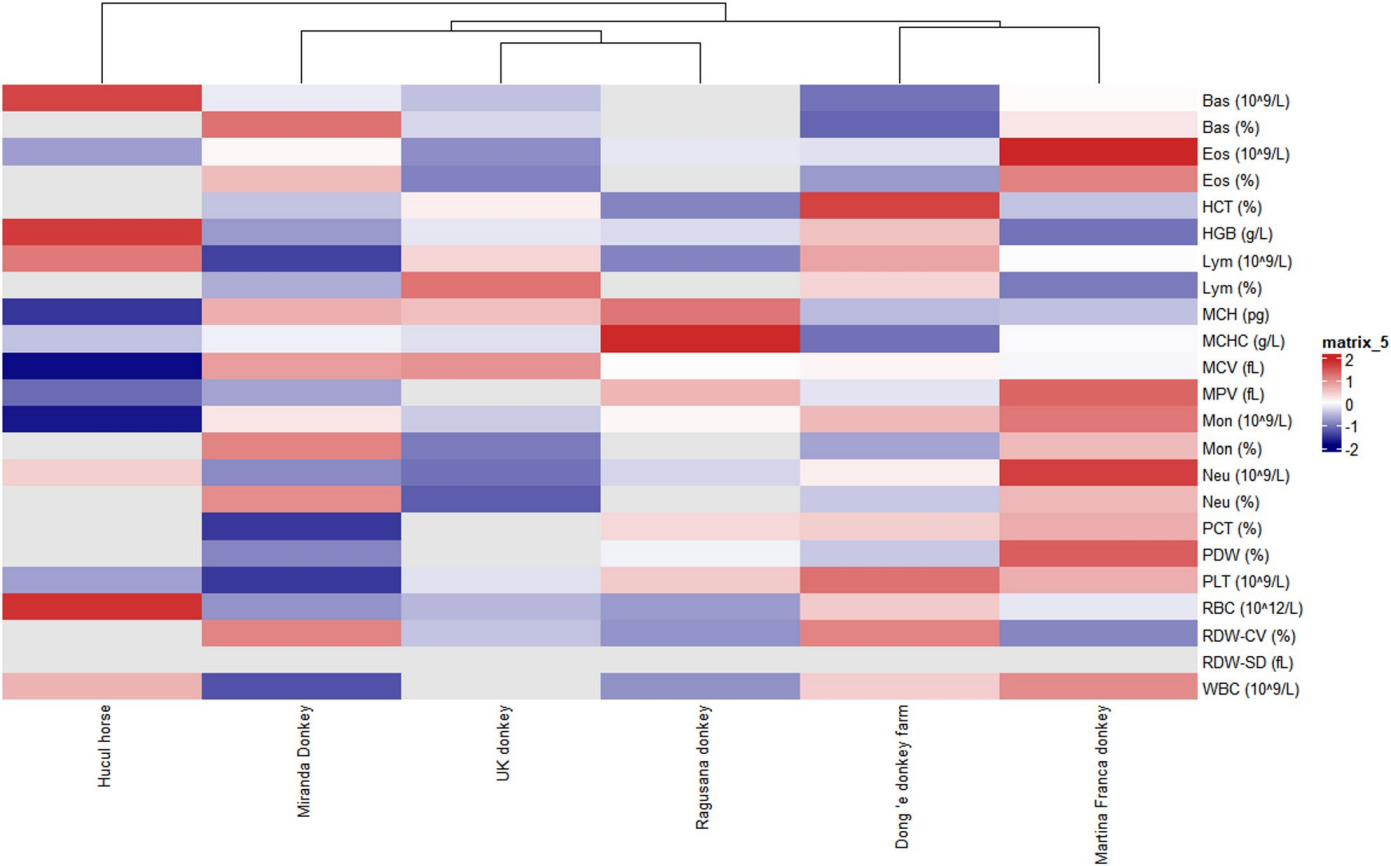
Cross-species comparison of reference intervals for hematological parameters

Using the hematological reference intervals established from the Dong’e donkey farm as the core reference framework, distinct stratification and systematic divergence were observed across species in hematological parameter distributions (Fig. 2). Overall, this reference system exhibited relatively stable intermediate levels in white blood cell count (WBC), red blood cell count (RBC), and platelet-related parameters. In the clustering structure, it formed an independent subbranch together with Martina Franca donkey, collectively belonging to the main donkey clade. Compared with this reference framework, Hucul horse showed markedly lower levels in erythrocyte-related parameters (e.g., HGB, RBC, and MCHC), representing the most pronounced interspecies divergence and the greatest deviation from the reference group. UK donkey, Ragusana donkey, and Martina Franca donkey exhibited consistently higher levels in leukocyte-related parameters (e.g., Eos, Neu, and WBC) relative to the reference intervals, indicating a systematic shift and suggesting structural differences in immune cell profiles compared with the Dong’e reference population. In addition, Miranda donkey showed generally lower values across multiple hematological parameters (including MCHC, MPV, and PLT), displaying a consistent low-expression pattern and a moderate separation from the reference system. For field veterinarians, the main clinical implication is that interpretation should be anchored to population- and management-specific donkey RIs rather than generalized equine values. In practice, this is especially important when evaluating mild increases in liver enzymes, differences in bilirubin, or apparently “abnormal” RBC/platelet values, because several of these findings may fall within the physiological range for Chinese local donkeys. The present intervals can therefore reduce overdiagnosis, improve case triage, and help distinguish normal adaptation to growth, sex, and management from genuine disease.

The data were obtained from [7, 8, 12, 36, 87], using the same methodology as described in Fig. 1.

Based on the findings above, the reference intervals established in this study provide a more accurate basis for interpreting clinical parameters in donkeys, reducing the risk of misdiagnosis associated with the use of horse-derived values. The cross-species comparison further improves the understanding of physiological differences among equids, thereby enhancing the accuracy of disease diagnosis and health assessment in field practice.

### Study limitations and potential sources of bias

Several limitations should be considered when interpreting these findings. First, the farms were not sampled randomly, and farm selection may have introduced population bias because each site differed in genetic background, housing, feeding strategy, and preventive management. Second, although all animals were clinically healthy at sampling, residual differences in management intensity, exercise load, season, workload, transportation, heat stress, and nutritional composition across farms may have contributed to between-farm variability. Previous studies have demonstrated that extreme environmental conditions, transportation, and workload-related stress can significantly affect hematological homeostasis in donkeys [88, 89]. In addition, sample handling procedures, seasonal variation, physical activity, body condition score, and individual nutritional status may also influence these parameters [90–93]. Third, the hematological reference intervals were derived from a single population (Dong’e donkey farm), which may limit their representativeness and generalizability to other Chinese donkey populations. Fourth, despite standardized procedures, analytical variability related to instrument performance, reagent lots, sample transport, and preanalytical handling cannot be fully excluded. Finally, although the overall sample size was relatively large, sex imbalance in certain subgroups, such as the Provincial Genetic Conservation Farm and Xinjiang Donkey Farm, may have affected the precision of RI estimation and the stability of distribution tails. Therefore, these results should be interpreted with caution, and future multicenter studies with broader geographic coverage and longitudinal validation are needed to confirm their robustness.

## Conclusions

This study established large-scale, CLSI-compliant hematological and serum biochemical reference intervals for Chinese local donkeys based on a multi-farm population. Although age- and sex-related differences were observed, they were not sufficient to justify partitioning. In contrast, farm origin emerged as the primary source of variation, underscoring the importance of population-specific reference intervals. Physiologically, the observed age-related changes were consistent with hepatic maturation, skeletal growth, and mineral homeostasis, while sex-related differences in RBC and platelet indices likely reflected hormonal modulation of erythropoiesis and thrombopoiesis. These results provide a useful baseline for improving diagnostic accuracy, supporting health monitoring, and facilitating future research in nutrition, genomics, and disease in donkey populations. For routine clinical use, the data support the adoption of Chinese local donkey-specific RIs in field practice, particularly when evaluating animals under distinct management systems or life stages.

## Materials and methods

### Animals

A total of 1,163 serum samples were collected from five donkey farms, including two national conservation farms (Dong’e and Wudi; *n* = 869), two provincial conservation farms (Yucheng and Zhongrun; *n* = 151), and one local production farm in Xinjiang (*n* = 143). For statistical analyses, samples were grouped into three management systems: national conservation, provincial conservation, and Xinjiang local production systems.The national conservation farm category comprised Dong’e (*n* = 828) and Wudi (*n* = 41) donkey farms. Notably, all age-related comparisons (juvenile vs. adult) were conducted exclusively using samples from the Dong’e farm to ensure population consistency. In addition, 935 whole-blood samples for hematological analysis were collected from the Dong’e farm. Due to limited serum volume and differences in sampling time points, individual animals included in serum biochemical and hematological analyses were not fully matched. However, all samples were derived from the same breeding system and managed under standardized husbandry conditions, ensuring biological comparability.

All animals were clinically evaluated by licensed veterinarians prior to sampling, and health status was determined through comprehensive physical examinations including assessment of mental state, appetite, behavioral responses, body condition score (BCS), rectal temperature, respiratory rate, and heart rate, together with farm records and on-site veterinary inspection, with only animals showing no evidence of infectious, metabolic, or systemic disease being included in the study [94]. Strict exclusion criteria were applied to ensure the quality of reference interval establishment, and animals were excluded if they presented abnormal body condition scores (BCS < 2.5 or > 4.0 on a 5-point scale), clinical signs of disease, pregnancy or lactation in females, evidence of parasitic infection, or a history of recent pharmacological treatment including antibiotics, anthelmintics, or hormonal agents, while individuals with overt clinical abnormalities or documented health disorders were not included [94]. All samples were collected in the morning under fasting conditions with minimal stress, and all animals were maintained under standardized management and routine veterinary preventive care, with information on age, sex, and origin recorded for subsequent stratified analyses.

For age-based analyses, donkeys were classified as adults (≥ 18 months of age) or juveniles (< 18 months of age) based on physiological maturity and dentition criteria established for equids.

### Blood Sample Collection and Laboratory Analyses

Blood samples were collected via jugular venipuncture from donkeys representing different conservation levels and production systems. Approximately 5 mL of whole blood was first collected into plain tubes for serum biochemical analysis, followed by an additional 5 mL collected into EDTA-K₂ anticoagulated tubes for hematological analysis. All samples were processed under standardized preanalytical conditions in the same central laboratory. Serum samples were allowed to clot fully at room temperature and were centrifuged on the day of collection at 3,000 × g. The separated serum was immediately aliquoted and stored at − 80 °C to ensure stability, and all biochemical assays were completed within one month after sampling. Hematological samples were stored at 4 °C and analyzed within 24 h of collection.

Serum biochemical analyses were performed using a Thermo Scientific Indiko automated biochemical analyzer (Thermo Fisher Scientific, USA) with reagents supplied by Ningbo Meikang Biotechnology Co., Ltd. The measured parameters included albumin (ALB), alkaline phosphatase (ALP), alanine aminotransferase (ALT), amylase (AMY), aspartate aminotransferase (AST), calcium (Ca), creatine kinase (CK), creatinine (CR), direct bilirubin (DBIL), iron (Fe), gamma-glutamyl transferase (GGT), glucose (GLU), lipase (LIP), magnesium (Mg), phosphorus (P), total bilirubin (TBIL), total cholesterol (TCH), triglycerides (TG), total protein (TP), and uric acid (UA).

Hematological analyses were conducted using a Mindray BC-5000 Vet automated hematology analyzer (Mindray, Shenzhen, China), calibrated for equine species. EDTA-anticoagulated whole blood was used for analysis. Hematological parameters included total white blood cell count (WBC, 10⁹/L); leukocyte differential counts expressed as both absolute values and percentages, including neutrophils (Neu, 10⁹/L; Neu, %), lymphocytes (Lym, 10⁹/L; Lym, %), monocytes (Mon, 10⁹/L; Mon, %), eosinophils (Eos, 10⁹/L; Eos, %), and basophils (Bas, 10⁹/L; Bas, %). Red blood cell indices included red blood cell count (RBC, 10¹²/L), hematocrit (HCT, %), hemoglobin concentration (HGB, g/L), mean corpuscular volume (MCV, fL), mean corpuscular hemoglobin (MCH, pg), mean corpuscular hemoglobin concentration (MCHC, g/L), red cell distribution width coefficient of variation (RDW-CV, %), and red cell distribution width standard deviation (RDW-SD, fL). Platelet parameters included platelet count (PLT, 10⁹/L), plateletcrit (PCT, %), mean platelet volume (MPV, fL), and platelet distribution width (PDW, %). All analyses were performed in the same laboratory using identical reagent batches to minimize analytical variation.

All procedures followed standard laboratory quality control protocols, including instrument calibration verification and the use of commercial quality control materials, to ensure analytical accuracy and stability. Samples showing severe hemolysis, lipemia, or icterus were excluded prior to analysis according to standard laboratory rejection criteria to minimize preanalytical interference. Blood cell and serum biochemical parameters were not used as preselection criteria; all samples were processed under identical standardized laboratory conditions using the same instruments, and all data were included in the final statistical analysis.

### Statistical analysis and reference interval establishment

All statistical analyses were performed using R software (v4.4.1) and IBM SPSS Statistics 22 [95]. Data distribution was assessed using skewness, kurtosis, Q–Q plots, and histograms. Normality was tested using the Shapiro–Wilk test. When necessary, Box–Cox transformation was applied to improve normality prior to parametric analyses. Continuous variables were summarized as median and interquartile range for non-normally distributed data, and as mean ± standard deviation for normally distributed data used in group comparisons.

Differences among donkey farms were analyzed using the Kruskal–Wallis test followed by Dunn’s post hoc test. Within each farm, reference intervals (RIs) were established in accordance with CLSI C28-A3 guidelines using a nonparametric approach based on the 2.5th–97.5th percentiles [96]. Outlier detection was performed using Tukey’s interquartile range (IQR) method, and removal was conducted only when supported by both statistical evidence and biological plausibility. Bootstrap resampling (1,000 iterations) was used to estimate 95% confidence intervals for RI limits, providing robustness assessment of the calculated reference intervals. The necessity for partitioning by sex and age was evaluated using the Harris & Boyd method, and partitioning was applied only when statistically justified; otherwise, data were pooled to ensure statistical robustness and clinical applicability [97]. For group comparisons within farms, one-way ANOVA or Welch’s ANOVA was used depending on homogeneity of variance assessed by Levene’s test. Post hoc multiple comparisons were performed using Tukey’s or Games–Howell tests as appropriate. P-values were adjusted using the Benjamini–Hochberg method, with adjusted P_adj < 0.05 considered statistically significant.

Hematological parameters from the Dong’e donkey farm, one of the National Genetic Conservation Farms, were analyzed using the same statistical framework, and reference intervals were established in accordance with CLSI C28-A3 guidelines. All statistical tests were two-sided, and *P* < 0.05 was considered statistically significant.

## Supplementary Information

Supplementary Material 1. Supplementary Table S1- Reference intervals for serum biochemical and hematological parameters.xlsx.

Supplementary Material 2. Supplementary Table S2-Harris and Boyd analysis for age and sex partitioning.xlsx.

## Data Availability

The datasets generated and analyzed in this study, including hematological and serum biochemical data from Chinese local donkeys, are available from the corresponding author upon reasonable request.
